# Therapeutic effects of dexamethasone-loaded hyaluronan nanogels in the experimental cholestasis

**DOI:** 10.1007/s13346-022-01132-7

**Published:** 2022-02-28

**Authors:** Sabina Di Matteo, Chiara Di Meo, Guido Carpino, Nicole Zoratto, Vincenzo Cardinale, Lorenzo Nevi, Diletta Overi, Daniele Costantini, Claudio Pinto, Elita Montanari, Marco Marzioni, Luca Maroni, Antonio Benedetti, Marco Viola, Tommasina Coviello, Pietro Matricardi, Eugenio Gaudio, Domenico Alvaro

**Affiliations:** 1 Department of Immunology, Bambino Gesù Childrens Hospital, IRCCS, Rome, Italy; 2 Department of Drug Chemistry and Technologies, Sapienza University of Rome, Rome, Italy; 3 Department of Movement, Division of Health Sciences, Human and Health Sciences, University of Rome “Foro Italico, Rome, Italy; 4 Department of Precision and Translational Medicine, Sapienza University of Rome, Rome, Italy; 5 Department of Medico-Surgical Sciences and Biotechnologies, Sapienza University of Rome, Rome, Italy; 6 Department of Biosciences, University of Milan, Milan, Italy; 7 Department of Anatomical, Forensic, Medicine and Orthopedic Sciences, Sapienza University of Rome, Rome, Italy; 8 Department of Gastroenterology and Hepatology, Università Politecnica Delle Marche, Ancona, Italy

**Keywords:** Nanogels, Dexamethasone, Hyaluronic acid, Cholestasis, Primary biliary cholangitis

## Abstract

A major function of the intrahepatic biliary epithelium is bicarbonate excretion in bile. Recent reports indicate that budesonide, a corticosteroid with high receptor affinity and hepatic first pass clearance, increases the efficacy of ursodeoxycholic acid, a choleretic agent, in primary biliary cholangitis patients. We have previously reported that bile ducts isolated from rats treated with dexamethasone or budesonide showed an enhanced activity of the Na^+^/H^+^ exchanger isoform 1 (NHE1) and Cl^−^/HCO_3_^−^ exchanger protein 2 (AE2) . Increasing the delivery of steroids to the liver may result in three beneficial effects: increase in the choleresis, treatment of the autoimmune or inflammatory liver injury and reduction of steroids’ systemic harmful effects. In this study, the steroid dexamethasone was loaded into nanohydrogels (or nanogels, NHs), in order to investigate corticosteroid-induced increased activities of transport processes driving bicarbonate excretion in the biliary epithelium (NHE-1 isoform) and to evaluate the effects of dexamethasone-loaded NHs (NHs/dex) on liver injury induced by experimental cholestatis. Our results showed that NHs and NHs/dex do not reduce cell viability in vitro in human cholangiocyte cell lines. Primary and immortalized human cholangiocytes treated with NHs/dex show an increase in the functional marker expression of NHE1 cholangiocytes compared to control groups. A mouse model of cholangiopathy treated with NHs/dex shows a reduction in markers of hepatocellular injury compared to control groups (NHs, dex, or sham group). In conclusion, we believe that the NHs/dex formulation is a suitable candidate to be investigated in preclinical models of cholangiopathies.

**Graphical abstract:**

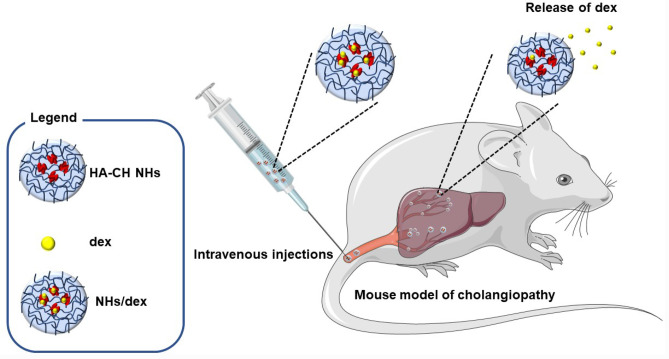

## Introduction

Biliary tree lined by cholangiocytes and peribiliary glands is primarily involved in certain cholestasis liver diseases named cholangiopathies. Cholangiopaties are major indications for orthotropic liver transplantation [1]. Intrahepatic biliary epithelium exert a pivotal barrier function against the harsh environment of the bile mainly due to an active bicarbonate excretion, the so-called protective “bicarbonate umbrella” [2].

The functioning of biliary extraction is driven by the differential potential across the cell membrane due to the ATP-dependent basal-lateral Na^+^/H^+^ pump. Actually, the excretion plays a crucial role in the intrahepatic biliary epithelium, mainly driven by the apical Na-independent Cl^−^/HCO_3_^−^exchanger, which is identified as isoform AE2 in rodents and humans, also know as solute carrier family 4 member 2 (SLC4A2) [3] and is functionally coupled with the cystic fibrosis transmembrane regulator (CFTR) [4]. The presence and functioning of two acid extruders, namely, the NHE-1 Na^+^/H^+^ exchanger isoform 1, also know as solute carrier family 9 member A1 (SLC9A1) and the Na^+^/HCO_3_^−^ cotransporter, balance the activity of the anion exchanger, as demonstrated in rodent cholangiocytes. This concert of flows is key in the regulation of intracellular pH (pHi) [4, 5]. As a key tool in liver homeostasis, the secretion of bicarbonate in the intrahepatic biliary epithelium is tightly regulated by ormones and neuropeptides [5], such as secretin, which induces choleresis by stimulating the Cl^−^/HCO_3_^−^ exchanger activity in cholangiocytes [5].

In human-acquired cholangiopathies, like primary biliary cholangitis (PBC) and primary sclerosing cholangitis (PSC), the expression and function of AE2 and secretin are decreasing, and this process induces choleresis [6].

In cystic fibrosis, the activity of the anion exchanger and bicarbonate excretion in bile is compromised because of a genetic CFTR defect. Budesonide, a corticosteroid with high receptor affinity and hepatic first pass clearance, improves the efficacy of UDCA-responder in the PBC patients [7, 8]. In PSC, prednisone exerts short-term beneficial effects when administered with UDCA, whereas budesonide had no effects [8]. Moreover, intrahepatic cholestasis of different origins may transiently improve after a short treatment with corticosteroids (corticosteroid whitewash) [6]. The intrahepatic biliary epithelium expresses glucocorticoid receptors (GcRs) and responds to dexamethasone by increasing bicarbonate excretion in bile [6]. A possible cause of the therapeutic effects of corticosteroids relies on an enhanced expression of the Na^+^/H^+^ (NHE1) and Cl^−^/HCO3^−^ (AE2) exchangers induced by corticosteroids [6].

One of the major limitations of steroids is their systemic adverse events, and even a corticosteroid with high receptor affinity and hepatic first pass clearance, like budesonide, may exert systemic unwanted effects, being particularly detrimental in patients with cholangiopathies, e.g. glucocorticoid-induced osteoporosis [9], or with advanced liver diseases, e.g. immune-suppression and infective risk [10].

Drug delivery systems (DDS) might be able to carry drugs (i.e. steroids), within the liver cells, increasing their therapeutic index [11] and avoiding issues associated with the prolonged systemic administration of high doses of steroids. Nowadays, several DDS have been studied, characterized and approved by FDA and EMA [11]. For example, liposomes have shown a good potential in improving the efficacy and tolerability of drugs of current use, e.g. antibiotics and anticancer drugs, even though attention must be paid on their stability during storage and administration in biological fluids [12, 13]. For improving stability issues, polymeric microparticles and nanoparticles were developed as an alternative [14]; both synthetic or natural biocompatible polymers were effective in encapsulating several kinds of drugs [15–17].

Recently, encapsulation of drugs into nanohydrogels (NHs) has emerged as a suitable alternative, able to enhance the therapeutic effectiveness of the entrapped drugs while minimizing their undesirable side effects [18]. NHs are nano-sized three-dimensional networks, with a soft nature, and able to absorb a large amount of water and to easily swell in aqueous media, thus showing the advantages of hydrogels at the nano-length scale. NHs represent a very versatile DDS since they are able to load and deliver both hydrophobic and hydrophilic low molecular weight drugs and polypeptides and genetic material [18].

Moreover, NHs composed of natural polymers (i.e. polysaccharides) offer the further advantages of being non-toxic, biocompatible and biodegradable [19]. Among polysaccharides, hyaluronic acid (HA) has been widely investigated in the development of biocompatible DDS as it naturally occurs in human body, being the major component of the extracellular matrix. HA is a linear non-sulfated glycosaminoglycan composed of alternating units of D-glucuronic acid and *N*-acetyl-D-glucosamine, linked together via alternating β-1,4 and β-1,3 glycosidic bonds. It is widely employed in cosmetic industry as well as in clinical applications (i.e. topical eye therapies, viscosupplementation and for the cutaneous treatment of inflammatory damages) [20]. HA can be easily functionalized with hydrophobic molecules (e.g. cholesterol or riboflavin) leading to amphiphilic derivatives able to spontaneously self-assemble in aqueous media, thus forming NHs [21–25]. Other interesting NHs were obtained using the gellan gum polysaccharide, functionalized with steroids (e.g. cholesterol or prednisolone) for the introduction of the hydrophobic moiety in the polymeric chains [26–28]. The advantage of using HA is related to its ability to recognize and bind some cell receptors, mainly CD44 receptor, which is constitutively expressed by the majority of hematopoietic, mesenchymal, epithelial and endothelial cells. Interaction of HA with its receptors leads to HA cellular internalization, where it triggers several intracellular events. In normal liver, only nonparenchymal liver cells (NPLC) express CD44, while in injured livers all the major NPLC increase CD44 expression. In addition, it is important to remind that 90% of HA is actively cleared by the liver [29]. Furthermore, in previous studies, it was demonstrated that fluorescent-labeled HA-CH NHs are mainly uptaken by the liver after intravenous injection, suggesting an important role of HA-based NHs in the pathogenesis of cholestasis [30].

The possibility to increase the delivery to the liver of steroids by means of a new kind of NHs can lead to three main positive effects: (I) the increase of choleresis; (II) the treatment of the autoimmune or inflammatory liver injury; and (III) the reduction of side effects of systemic corticosteroids [6, 9, 10]. Hence, in this study, the steroid dexamethasone was loaded into self-assembled hyaluronan-cholesterol NHs with the aim to investigate corticosteroid-induced enhanced of transport processes driving bicarbonate excretion in the biliary epithelium (NHE-1 isoform) and to evaluate the effects of dexamethasone-loaded NHs (NHs/dex) on liver injury induced by experimental cholestasis.

## Materials and methods

### Chemicals

Hyaluronan tetrabutylammonium salt (HA^−^TBA^+^) was purchased by Contipro (Dolní Dobroucˇ, Czech Republic) as HA^−^Na^+^ salt (Mη = 2·10^5^) and exchanged to TBA^+^ form using Dowex resins; Cholesterol (CH) was a Carlo Erba product; dexamethasone (dex), 4-bromobutyric acid, 4-(dimethylamino) pyridine (DMAP), N (3-dimethylaminopropyl)-N′-ethylcarbodiimide (EDC) and N-methylpyrrolidone (NMP) were purchased from Merck. Other chemicals were reagent grade and used without further purifications.

### Human primary cell cultures and tissue sourcing

Human extrahepatic biliary tree surgical resections were obtained from the “Paride Stefanini” Department of General Surgery and Organ Transplantation, Sapienza University of Rome, Italy [31, 32]. All donors were adults between the ages of 19 and 73, and they signed the written informed consent for research purposes obtained from our transplant program.

The research protocols follow the Good Manufacturing Practice and were approved by the Institutional Review Board of the Sapienza University of Rome, Italy, and by the Ethic Committees of Policlinico Umberto I of Rome, Italy.

### Cell isolation, cell cultures, media and solutions

Human biliary stem/progenitor cells (hBTSCs) have been isolated from human organs as previously described [31, 33–36]. hBTSCs primary cells were cultures in Kubota’s Medium (KM) and differentiated in cholangiocytes by hormonally defined medium as previously described [31].

### hBTSCs differentiation

Serum-free Kubota’s Medium was supplemented with calcium (final concentration 0.6 mM), copper (10^−12^ M) and 20 ng/mL basic fibroblast growth factor (bFGF, Merck, Germany) and referred to as modified Kubota’s Medium (MKM, Merck). HDM for cholangiocyte differentiation (CM, Merck) [31, 34]: MK3M supplemented with 20 ng/mL vascular endothelial cell growth factor (VEGF, Merck) 165 and 10 ng/mL human hepatocyte growth factor (HGF, Merck).

### Immortalized cell culture

H69 cell line human is a commercial cholangiocyte cell line grown in H69 medium as described in a previous work [37].

### MTS cell proliferation assay

Cell proliferation was evaluated by MTS assay (CellTiter 96 AQueous MTS Reagent Powder, PROMEGA, #TB245, Italy). Approximately 5·10^4^ of H69 cells and 2·10^5^ of hBTSCs-CM were seeded into 96-well plates in 150 μL of culture medium. The MTS assay was performed as described in detail in a previous paper [37, 38].

### Population doubling time (PDT)

The proliferation rate was analysed on the populations, seeded in 6 multi-well plates at the density of 8·10^4^ cells/well for H69 and 4·10^5^ cells/well for hBTSCs-CM. After experiments, cells were detached by Tripsine, collected, stained with trypan blue (Merck). The PDT was calculated in the phase exponential growth by the following Eq. (1) [39]:

1 $$\text{PDT}=\frac{\log10\Delta \text{T}}{\log10\left({\text{N}}_{3\text{d}}\right)}-\log10({\text{N}}_{1\text{d}})$$

N3d is the cells number at day 3, and N1d is the cell number at day 1. The mean of the cell number was calculated on six experimental samples for each condition, and the resulting time was expressed in day.

### Cell viability

Cell viability was determined by trypan blue exclusion assay (Merck). The cells stained in blue were dead; the viable cells did not stain. This dye was used at 1:1 v/v with the cell buffer. The cell count was carried out using FAST-READ 102 (Merck). Cell viability was calculated after 72 h of treatments.

### Quantitative reverse-transcription polymerase chain reaction (RT-qPCR) analysis

Total RNA was extracted from cell cultures by using TRIzol (Thermo Fisher Scientific, MA, USA) and the procedures of Chomczynski and Sacchi. Gene expression was determined by RT-qPCR described in a previous paper [37, 38]. NHE-1 gene expression level was normalized to the expression of GAPDH (housekeeping gene): GAPDH forward, 5-AGCCACATCGCTCAGACAC -3 and reverse, 5-GCCCAATACGACCAAATCC-3; NHE-1 forward, 5-CTC ATCTGTGCCTGTCTGTCC and reverse, 5-TCTGATGTCA CAGTCTTCGAGCAA-3.

### Murine model

Male and female C57BL6/J background mice were used as an in vitro model for this study.

The mice were 4–5 weeks old (mean body weight 25 g, purchased from Charles River Laboratories, Wilmington, MA), and they were maintained under standard conditions according to the institutional guidelines for animal care. The working and experimental protocols with animals were approved by Sapienza University of Rome Animal Welfare and Ethical Review Body.

To induce bile duct injury, mice were given 0.1% 3,5-diethoxycarbonyl-1,4-dihydrocollidine (DDC) mixed with Rat & Mouse No1 Maintenance (RM1) diet (Special Diet Services), for 28 days. After DDC diet, mice were given normal chow and drinking water for the successive 17 days.

### ELISA

AlkP and ALT levels in murine sera were analysed using the Human ALKP ELISA Kit (Thermo Fisher Scientific, #EH19RB) and Human ALT ELISA Kit (#ab234578, Abcam, UK), respectively, following the manufacturer’s specifications.

Results were expressed as U/L.

### Histology and immunohistochemistry (IHC) in situ

For hematoxylin and eosin stains of murine livers and Sirius red stains and immunohistochemistry in murine livers, we used the protocols already described [38, 40].

For cytokeratin 19 (CK19) staining, we used primary antibody anti-cytokeratin 19 antibody (RCK108, ab9221, Abcam).

The number of positive cells was automatically calculated by an algorithm on the entire section, and, then, a semi-quantitative scoring system was applied (0 =  < 5%; 1 = 6–10%; 2 = 11–30%; 3 = 31–50%; 4 =  > 50%).

### Preparation and characterization of dexamethasone NHs formulation

Hyaluronan-cholesterol nanohydrogels (HA-CH NHs) were prepared as already described [21, 41]. Briefly, the synthesis of the amphiphilic HA-CH derivative was carried out through the functionalization of HA^−^TBA^+^ with the Br-butyric derivative of cholesterol (CH-Br) at 15% mol_CH_/mol_HA_ [41]. Specifically, 200 mg of HA^−^TBA^+^ was dissolved in 10 mL of NMP, then 26 mg of CH-Br solubilized in 2 mL of NMP was added to HA^−^TBA^+^ solution, and the reaction was allowed to proceed for 48 h at 38 °C under vigorous magnetic stirring. Then, 1 mL of an aqueous saturated NaCl solution was added drop by drop to the mixture, which was left under stirring for 30 min to allow the replacement of TBA^+^ ions with Na^+^ ions. The product was then precipitated by adding 40 mL of acetone and left for 1 h at 4 °C. The supernatant was removed, and the product dispersed in bi-distilled water and finally purified by dialysis (Visking tubing, MW cut-off: 12,000–14,000) against bi-distilled water until the conductivity was lower than 1.5 μS [41]. The final suspension was frozen in liquid nitrogen and freeze-dried by using a “Modulyo 4 K” Edwards High Vacuum instrument, equipped with an Edwards pump, operating at 0.2 atm and at − 40 °C. HA-CH was recovered as a white lyophilized sample (yield: 90%) and analysed by FT-IR (Perkin-Elmer Spectrum One instrument equipped with ATR system) recording 32 scans in the range 650–4000 cm^−1^ in ATR mode.

Empty NHs, formed by the self-assembling of HA-CH molecules, were prepared as already described in detail in previous works [21, 42] by an autoclave standard sterilization cycle (20 min at 121 °C and 1.10 bar) on a polymer suspension in bi-distilled water (1 mg/mL, 30 mL), using a Juno Liarre autoclave 230 Vac, 50/60 Hz, 12A, 2000 W.

Dexamethasone-loaded NHs (NHs/dex) were prepared in a similar way: first, a thin film of the drug (20 mg) was prepared in a bottle by evaporating under vacuum 5 mL of a dex solution in acetone, followed by its hydration with a HA-CH suspension (1 mg/mL, 30 mL) for 1 h, under vigorous magnetic stirring, before being autoclaved as described above. The unloaded dex was removed from the NHs/dex suspension by centrifugation (20 min, 5000 rpm, 20 °C), solubilized with an excess of ethanol and analysed by HPLC using a Varian System 210 equipped with a Varian ProStar 325 UV–Vis detector and a Knauer C18 column, 250 mm × 4.6 mm (Eurospher II, 100–5 C18). The mobile phase, at the isocratic flux of 1 mL/min, consisted of a mixture of H_2_O: ACN (50:50), and the injection volume was 20 µL. The unloaded drug was quantified by using a dex calibration curve in ethanol at the concentration range of 25–250 μg/mL (λ = 239 nm, *R*^2^ = 0.9995).

The encapsulation efficiency (EE%) and drug loading (DL%) values of NHs/dex were calculated using the following equations:

2 $$\% \; \text{EE}=\frac{\text{dex}_\text{i}- \text{dex}_\text{u}}{\text{dex}_\text{i}} \times 100$$

3 $$\% \text{ DL}=\frac{\text{dex}_\text{l} }{\text{p}+ \text{dex}_\text{l}} x 100$$

where *dex*_*i*_ is the starting dex amount, *dex*_*u*_ is the unloaded drug, *dex*_*l*_ = *dex*_*i*_* − dex*_*u*_ is the loaded dex and *p* is the weight of NHs.

NHs and NHs/dex mean hydrodynamic diameter and polydispersity index (PDI) were measured by dynamic light scattering (DLS) by using a Submicron Particle Sizer Autodilute Model 370 (Nicomp, CA, USA). The ζ-potential of the NHs suspensions was measured in double-distilled water by using a Malvern NanoZetaSizer apparatus (Malvern Instruments, UK), equipped with a solid state 5 mW HeNe laser (λ = 632.8 nm) at a scattering angle of 173°. The electrophoretic mobility of the samples was converted in ζ‐potential by using the Smoluchowski equation.

For the in vivo experiments, both the osmolarity and the pH of the NHs and NHs/dex formulations were adjusted to 290 mOsm/L and to pH = 7.4 by addition of a suitable amount of glycerol (final concentration: 2% w/V) and simulated intestinal fluid (SIF) buffer (final concentration: 10 mM, pH = 7.4), respectively. The stability of the NHs and NHs/dex suspensions was checked by DLS over 2 weeks by keeping the samples at 4 °C. Moreover, for a long-term storage, the suspensions were added to a dextrose solution to obtain a final concentration of 0.5% w/V and subsequently freeze-dried; after the re-constitution of NHs with a suitable amount of water (*r*-NHs and *r-*NHs/dex), their size and PDI were studied by DLS analysis, and the stability of the systems (Gly 2% w/V, SIF 10 mM pH = 7.4, T = 37 °C) was checked for 3 days, in simulated physiological conditions.

### Statistical analysis

Statistical analyses were conducted using the paired or unpaired Student’s *t* test, and Bonferroni correction for multiple comparisons was used as appropriate. The analysis of the variance (ANOVA) were performed for multiple comparisons. Statistical significance was set to a *p* value < 0.05.

## Results

### Characterization of NHs/dex formulation

**Fig. 1:**
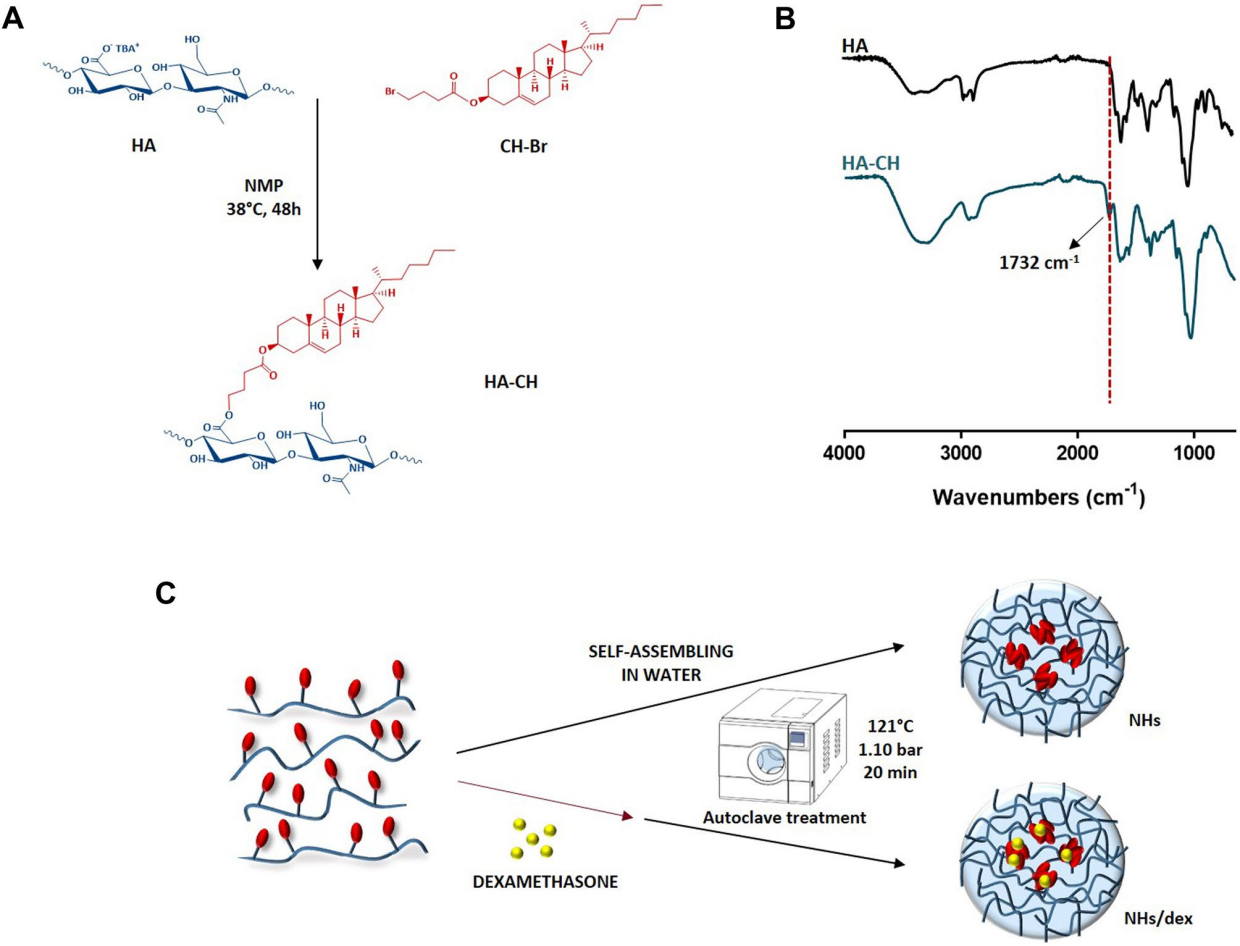
Scheme of the synthesis of HA-CH derivative (**A**); FT-IR spectra of HA^−^Na^+^ (black line) and HA-CH derivative (blue line): the band of the ester bonds at 1732 cm^−1^ is underlined (**B**); schematic representation of the preparation of NHs and NHs/dex samples by the autoclaving process (**C**)

Polysaccharides can be easily functionalized with small molecules in order to tune their physico-chemical properties, such as their solubility. To this purpose, in this work HA carboxyl groups were esterified with CH-Br moieties to form HA-CH which is an amphiphilic derivative of HA. The reaction was carried out in NMP, at 38 °C for 48 h (Fig. 1A), as the TBA^+^ salt of HA allowed the polymer solubilization in the aprotic polar solvent. At the end of the reaction, the product, as Na^+^ salt, was purified by precipitation with pure acetone, followed by an extensive dialysis against distilled water, and finally recovered by freeze-drying in high yield (∼90%).

The structure of the HA-CH derivative was confirmed by FT-IR spectroscopy. Figure 1B shows the FT-IR ATR spectra of both HA^−^Na^+^ (black line) and HA-CH derivative (blue line): the strong stretching absorption at 1732 cm^−1^ might be ascribed to the C = O stretching of the ester bonds formed.

Then, an aqueous suspension of HA-CH (1 mg/mL) was treated with a sterile autoclaving cycle (121°C, 20 min, 1.1 bar) to produce self-assembled NHs (Fig. 1C). For NHs/dex, the same process was carried out in the presence of a dry film of the drug, leading simultaneously to the formation and the loading of sterile NHs (Fig. 1C). The unloaded dex was removed from the NHs/dex suspension by a mild centrifugation, and the E.E.% of the formulation was quantified by using HPLC.

Specifically, the % EE and % DL, defined by Eq. 2 and Eq. 3, were 23 ± 2% and 13 ± 1%, respectively, corresponding to a NHs drug concentration of 154 ± 12 µg/mL.

The NHs/dex formulation showed an average size of 222 ± 7 nm, as measured by DLS, with a low PDI (0.14 ± 0.03); the ζ-potential net value of the NHs/dex suspension, assessed by ZetaSizer analysis, was high enough to ensure a good stability of the nano-formulation (− 49 ± 3 mV).

**Fig. 2:**
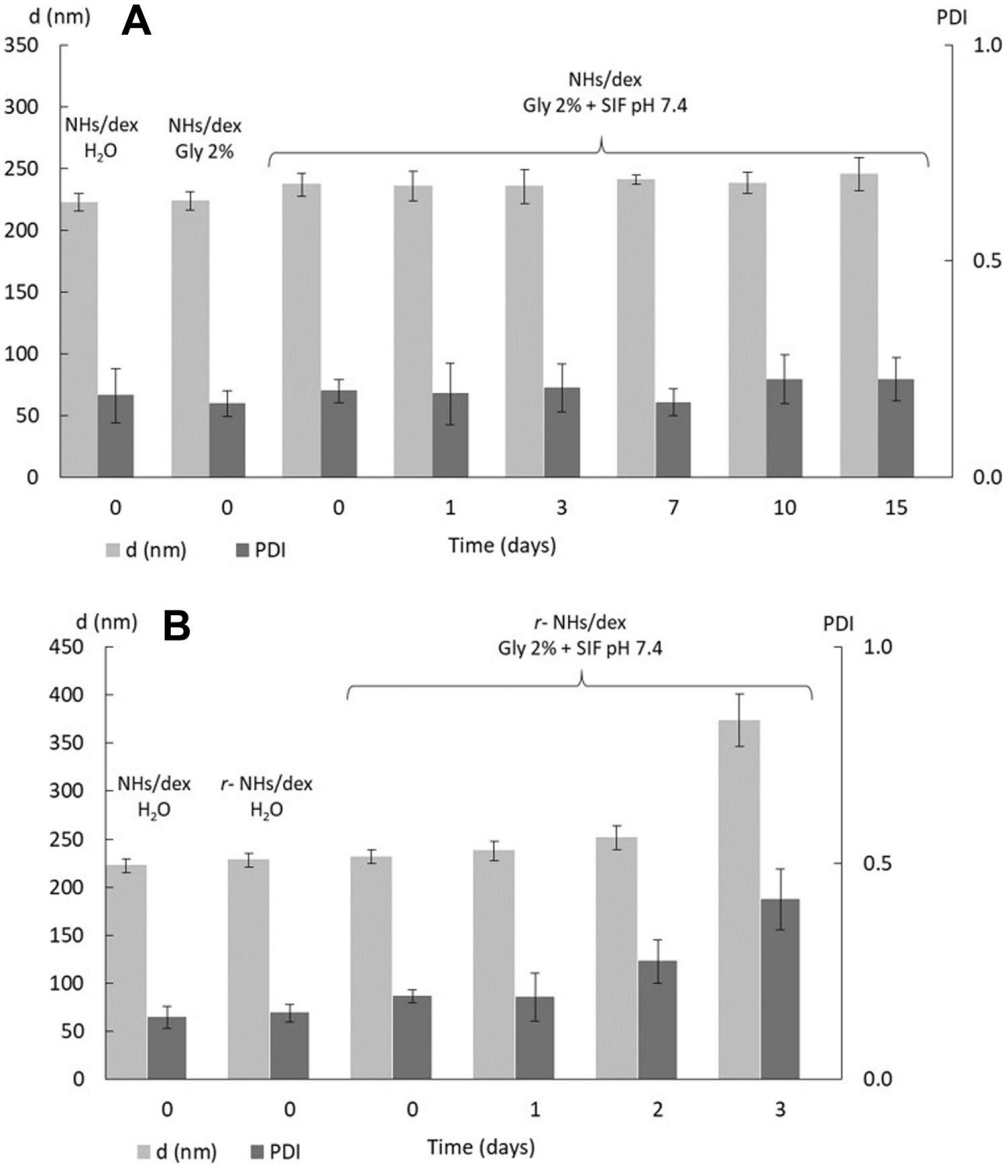
(**A**) Stability of NHs/dex in water and in gly/SIF conditions at 4 °C (*n* = 3); (**B**) Stability of NHs/dex after reconstitution from the lyophilized product in gly/SIF conditions at 37 °C (*n* = 3)

For the in vitro and in vivo experiments, both the pH and osmolarity values of NHs and NHs/dex suspensions were adjusted by the addition of appropriate amounts of SIF buffer at pH = 7.4 and glycerol, respectively. The stability of the NHs/dex formulation in such conditions was monitored at 4 °C, as shown in Fig. 2A: no significant increase in both size and PDI values was noticed over 2 weeks. Moreover, to assess a long-term storage of the formulation, the freeze-drying process was tested on NHs/dex aqueous suspension after the addition of a dextrose solution (final concentration 0.5% w/V) as cryoprotectant. After sample reconstitution with a suitable amount of bi-distilled water (*r-*NH/dex) and the addition of gly/SIF, the stability of *r-*NHs/dex was monitored at 37 °C for 3 days, showing an increase in both size and PDI at the day 3, as shown in Fig. 2B.

### Activity on metabolism and replication of human cholangiocytes in vitro

**Fig. 3:**
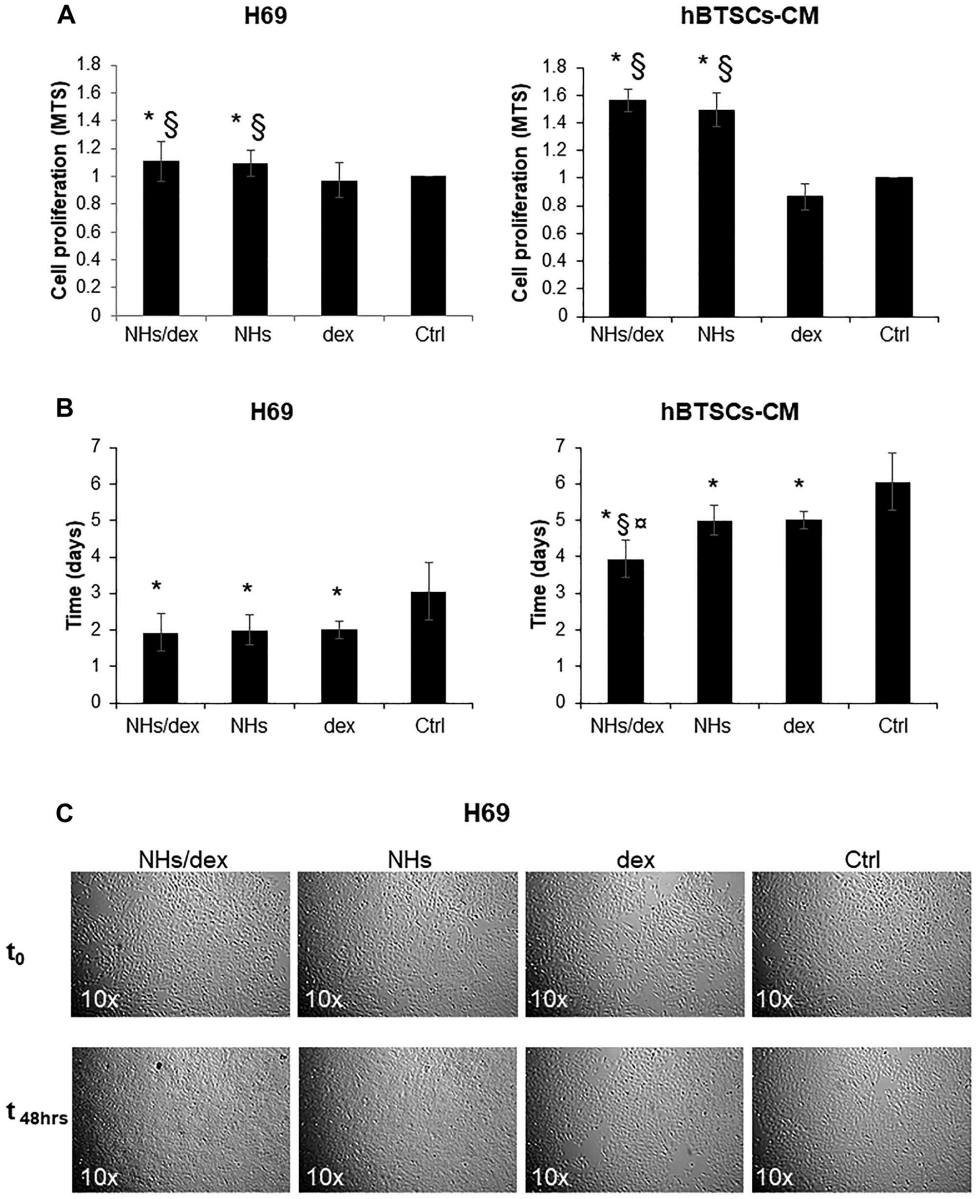
(**A**) H69 and hBTSCs-CM primary cell cultures were exposed for 48 h to NHs/dex, NHs, dex or medium only (Ctrl). Cell proliferation was evaluated by MTS assay and expressed as ratio compared to controls. NHs/dex treatment significantly increases cell proliferation compared to dex and Ctrl. Data represent the mean ± SD of *N* = 5 independent experiments. *p < 0.05 vs Ctrl; § *p* < 0.05 vs dex. (**B**) PDT was calculated as the time (days) required by cell cultures to duplicate their cell number. NHs/dex markedly reduces the PDT. Data represent the mean ± SD of *N* = 5 independent experiments. *p < 0.05 vs Ctrl; § *p* < 0.05 vs dex; ¤ *p* < 0.05 vs NHs. (**C**) Optical microscopy pictures of H69 exposed to NHs/dex, NHs; dex or medium only (Ctrl) for 48 h; from the images, it is possible to observe the cell density increase (vs controls), and no morphological changes can be observed in the cells. Magnification 10 × , representative images of *N* = 5 independent experiments

The effects of NHs/Dex were evaluated in human immortalized normal cholangiocytes cell cultures (H69) and in hBTSCs obtained from liver organ donors differentiated in mature cholangiocyte (hBTSCs-CM). First, the sensitive quantification of viable and metabolically active cells by the colorimetric and high throughput method MTS assay was conducted. H69 and hBTSCs-CM were seeded on plastic cultured in their specific media. Thereafter cells were incubated for 72 h with several conditioned media with NHs/Dex, NHs, or dex, or left in basal medium used as control (Ctrl). Results of MTS assay showed in H69 a significant, although not dramatic, increase of cell proliferation in NHs/Dex vs Dex and vs Ctrl or in NHs vs dex and vs Ctrl (Fig. 3A). Similar results but more evident were observed in hBTSCs-CM. An analysis of the population doubling time was conducted by counting viable cells through the trypan blue exclusion test at different time points. In H69 number of viable cells (vitality assay) growth in conditioned media (NHs/Dex, NHs, or Dex) showed a trend through an increase according to the MTS assay, confirming the non-toxicity of NHs or NHs/Dex (not shown). Accordingly, the PDT showed a trend through a decrease in all conditioned media (NHs/Dex, NHs, or Dex) compared to cell growth in control medium (Ctrl, *p* > 0.05) (Fig. 3B). Similar results were observed in hBTSCs-CM cultures for MTS assay and vitality assay (Fig. 3A). hBTSCs-CM treated with NHs/dex showed a statistically reduced PDT compared to all other groups (Fig. 3B). The observation of the cells under an optical microscope (Fig. 3C) confirms the data described above with a moderate increase in cell density. No morphological changes were observed in the cells which showed a normal proliferative rate.

### Activity on the expression of choleretic gene NHE-1 in vitro

In cholangiocytes, physiologic choleresis process is maintained by the acid extruder NHE-1. The NHE-1 expression was evaluated in H69 cell cultures and in hBTSCs-CM primary cultures exposed to NHs/dex, NHs, dex, or normal medium (Ctrl) for 72 h by RT-qPCR.

**Fig. 4:**
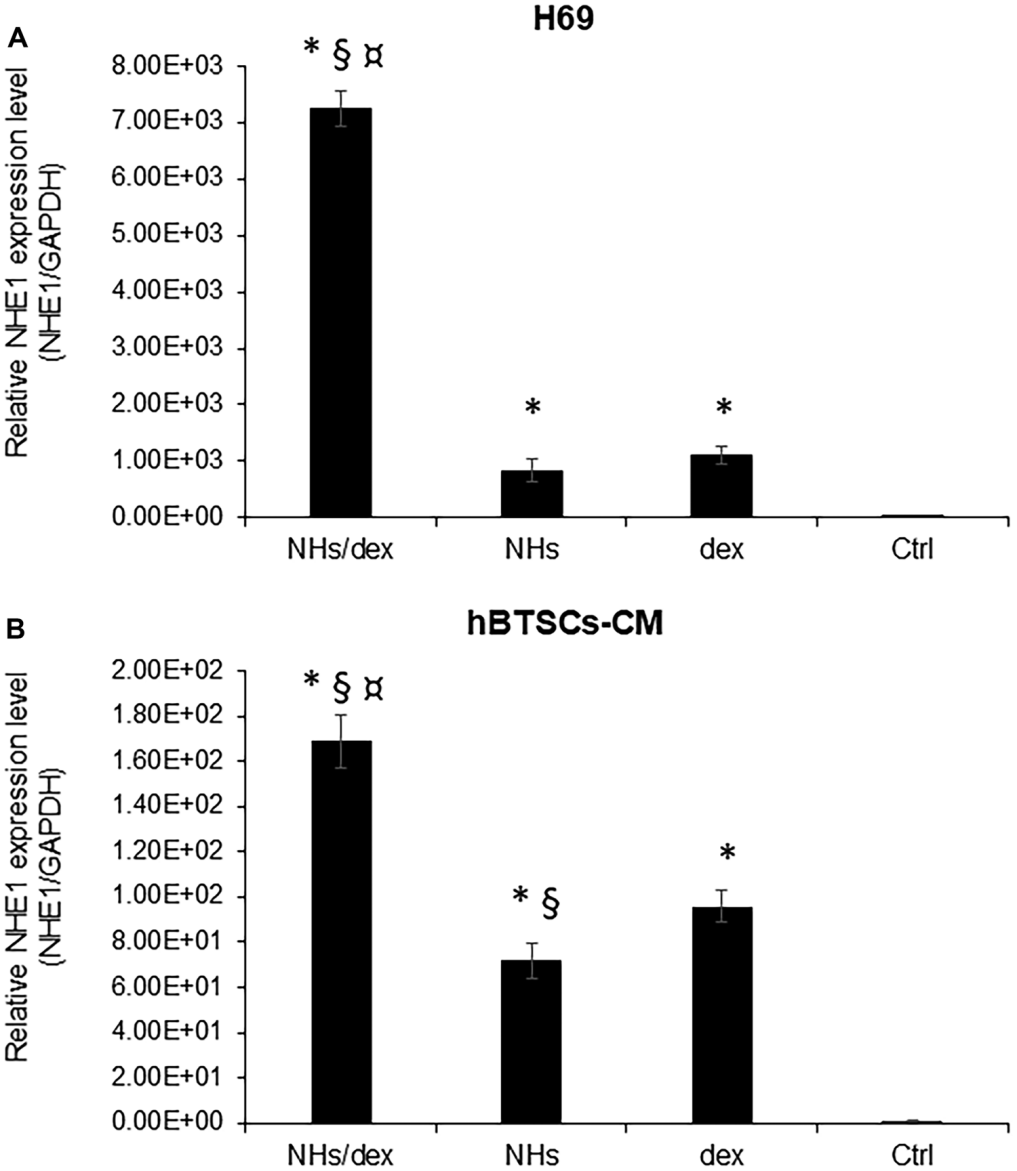
The NHE-1 relative gene expression was analysed by RT-qPCR in H69 (**A**) and hBTSCs-CM (**B**) primary cultures after 48 h of culture with NHs/dex, NHs, dex or medium only (Ctrl) for 48 h and normalized to the expression of GAPDH (housekeeping gene). In both H69 and hBTSCs-CM, the gene expression of NHE-1 was significantly increased by NHs/dex compared to other treatments and Ctrl. Data represent the mean ± SD of *N* = 3 independent experiments. *p < 0.05 vs Ctrl; § *p* < 0.05 vs dex; ¤ *p* < 0.05 vs NHs

In the H69, the NHE-1 expression in NHs/dex group (7.24·10^3^ ± 3.00·10^2^) was significantly higher than NHs group (8.25·10^2^ ± 2.00·10^2^) dex group (1.11·10^3^ ± 1.50·10^2^) and control group (Ctrl, normalized to unity, *p* < 0.05). There is no statistically significant difference between the NHs and dex groups (*p* > 0.05) and between these and the Ctrl (*p* > 0.05) (Fig. 4A).

In hBTSCs-CM, NHE-1 expression increased in all experimental conditions compared to the basal medium. In NHs/dex group, the gene expression of NHE-1 (1.69·10^2^ ± 1.20·10^1^) is much higher and statistically significant than in all other groups (*p* < 0.05 vs others) (Fig. 4B).

### Activity on liver injury in vivo in a mouse model of cholangiopathy

The biliary epithelium is the specific target of damage in a group of chronic cholestatic liver diseases, the cholangiopathies, which represent a major indication for liver transplantation. To induce bile duct injury, mice were given 0.1% DDC mixed with a maintenance diet for 28 days. Thereafter, the treatment with DDC was interrupted, and mice were randomly divided into five groups (*N* = 6 per group) and were subjected for 17 days to daily intravenous injections (tail vein), respectively, of saline (DDC Ctrl group), dex (DDC + dex group, 30.8 µg per injections), NHs alone (DDC + NHs group, 30.8 µg per injections), or NHs loaded with dex (DDC + NHs/dex group, 30.8 µg per injection). A control group in which mice were never treated with DDC, but always received a normal diet, was used as a sham group (Normal Ctrl group, *N* = 6).

**Fig. 5:**
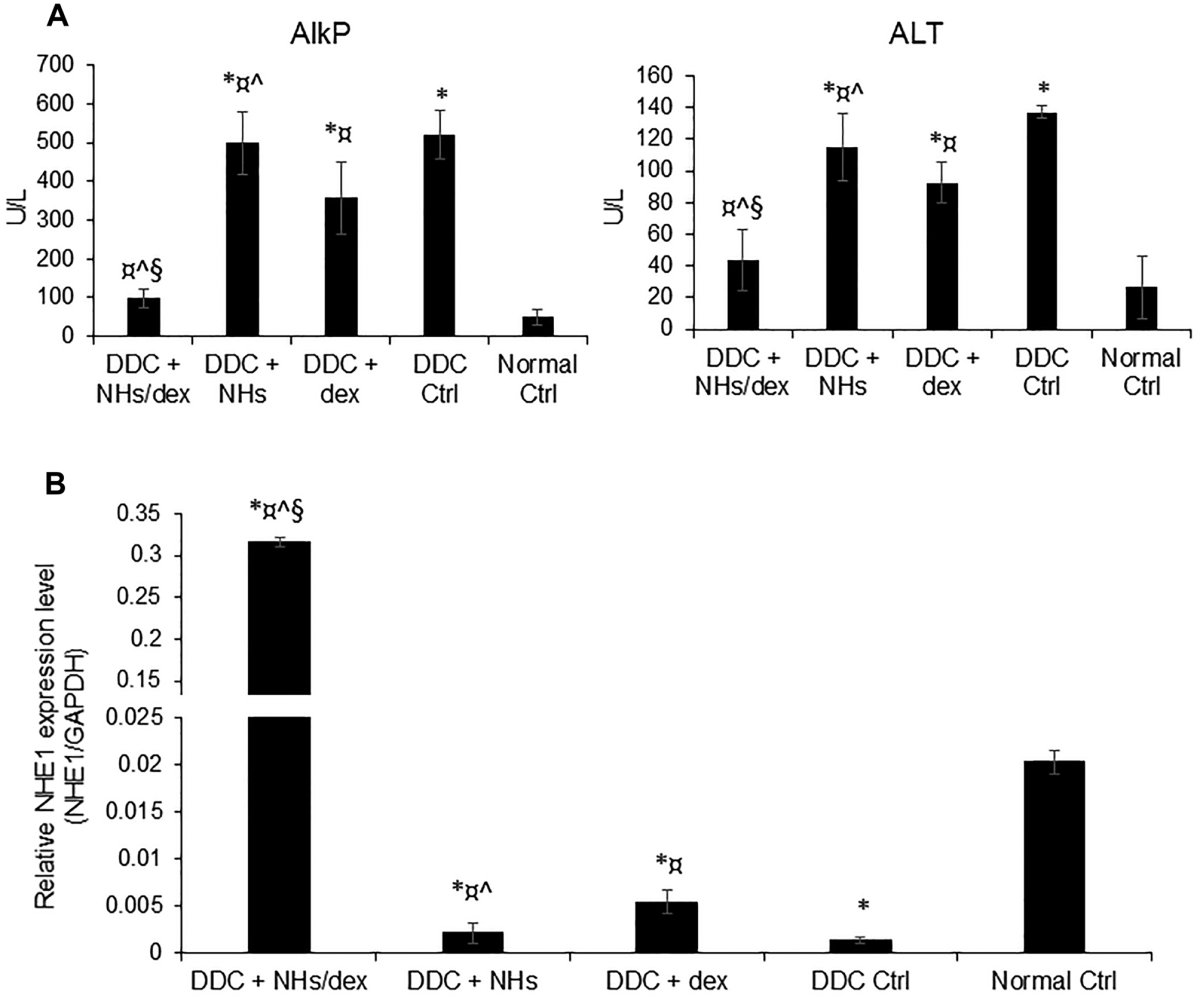
(**A**) Analysis of AlkP and Alt levels on murine sera on the day of sacrifice by ELISA. Mice treated with DDC to induce bile duct injury and subsequently treated with NHs/dex (DDC + NHs/dex) did not have significant AlkP and ALT levels compared non-DC mice that did not receive any treatment (Normal Ctrl group). Mice treated with DDC + NHs, DDC + dex and DDC Ctrl groups showed significant high levels of AlkP and ALT compared to Normal Ctrl group. Furthermore, the DDC + NHs/dex group had AlkP and ALT statistically lower compared to the other DDC groups. Data represent mean ± SD of *N* = 6 independent experiments. *p < 0.05 vs Normal Ctrl; ¤ *p* < 0.05 vs DDC Ctrl; ^ *p* < 0.05 vs DDC + Dex; § *p* < 0.05 vs DDC + NHs. (**B**) NHE-1 relative gene expression was analysed on murine liver sections by RT-qPCR and normalized to the expression of GAPDH (housekeeping gene). NHE-1 gene expression in DDC + NHs/dex mice was statistically higher than in all other experimental groups. Data represent the mean ± SD of *N* = 6 independent experiments. *p < 0.05 vs Normal Ctrl; ¤ *p* < 0.05 vs DDC Ctrl; ^ *p* < 0.05 vs DDC + Dex; § *p* < 0.05 vs DDC + NHs

At the end of the experiments, the serum of the mice was collected and analysed for markers of hepatocellular injury, e.g. ALT, and cholestasis, e.g. alkaline phosphatase necrosis (AlkP) (Fig. 5A). The mice of Normal Ctrl group showed a very low value of both markers. As far as AlkP, confirming the biliary nature of the injury, mice subjected to DDC diet and saline injection (DDC Ctrl group) showed a dramatic increase of cholestatic marker (520 ± 63 U/l; *p* < 0.05, *N* = 3) compared to mice in Normal control group. The groups of DDC + dex or DDC + NHs similarly experienced an increase of cholestatic marker compared to mice in Normal control group (*p* < 0.05), and only the treatment with dex induced a slight, although not significant, reduction of AlkP. Notably, instead, the treatment with NHs/dex induced a dramatic decrease of AlkP (DDC + NHs/dex, 96.3 ± 12 UI) with respect to the DDC Ctrl (*p* < 0.05), and with respect to the dex and NHs groups (*p* < 0.05), and almost induced a complete normalization of the cholestatic maker. As far as transaminases ALT, confirming the well known secondary cholestatic-induced hepatocellular injury, mice subjected to DDC diet and saline injection (DDC Ctrl) showed a dramatic increase of the ALT levels (137 ± 5 UI; *p* < 0.05, *N* = 3) with respect to the Normal Ctrl mice. The groups of dex or NHs alone similarly experienced an increase of cholestatic marker with respect to the Normal Ctrl group (*p* < 0.05), and only the treatment with dex induced a significant reduction of ALT compared to the DDC Ctrl group (*p* < 0.05). Notably, instead, the treatment with NHs/dex induced a dramatic decrease of ALT (43.5 ± 18 UI, *N* = 3) compared to DDC Ctrl group (*p* < 0.05), and with respect to the dex and NHs groups (*p* < 0.05), and almost induced a complete normalization of the necrotic damage maker.

### Activity on the expression of choleretic gene NHE-1 in vivo in a mouse model of cholangiopathy

The mice liver, of the groups described above, was collected on the day of sacrifice on the seventeenth day of treatment, and the NHE-1 gene expression was evaluated by RT-qPCR (Fig. 5B).

Mice subjected to DDC diet and saline injection (DDC + Ctrl) showed a dramatic decrease of NHE-1 gene expression compared to mice under control diet (Normal Ctrl group) (*p* < 0.05). The groups of DDC + dex or DDC + NHs similarly experienced a reduction of the NHE-1 expression with respect to the control diet (*p* < 0.05), and only the treatment with dex induced significant increase of the gene expression with respect to the DDC Ctrl mice (p < 0.05). Notably, instead, the treatment with NHs loaded with dex (DDC + NHs/dex) induced a dramatic increase of the NHE-1 expression compared to DDC Ctrl group (*p* < 0.05), and with respect to the DDC + dex and DDC + NHs groups (*p* < 0.05). The value resulted even increased with respect to the expression in normal liver (Normal Ctrl) (*p* < 0.05).

### Activity on liver tissue damage in vivo in a mouse model of cholangiopathy

**Fig. 6:**
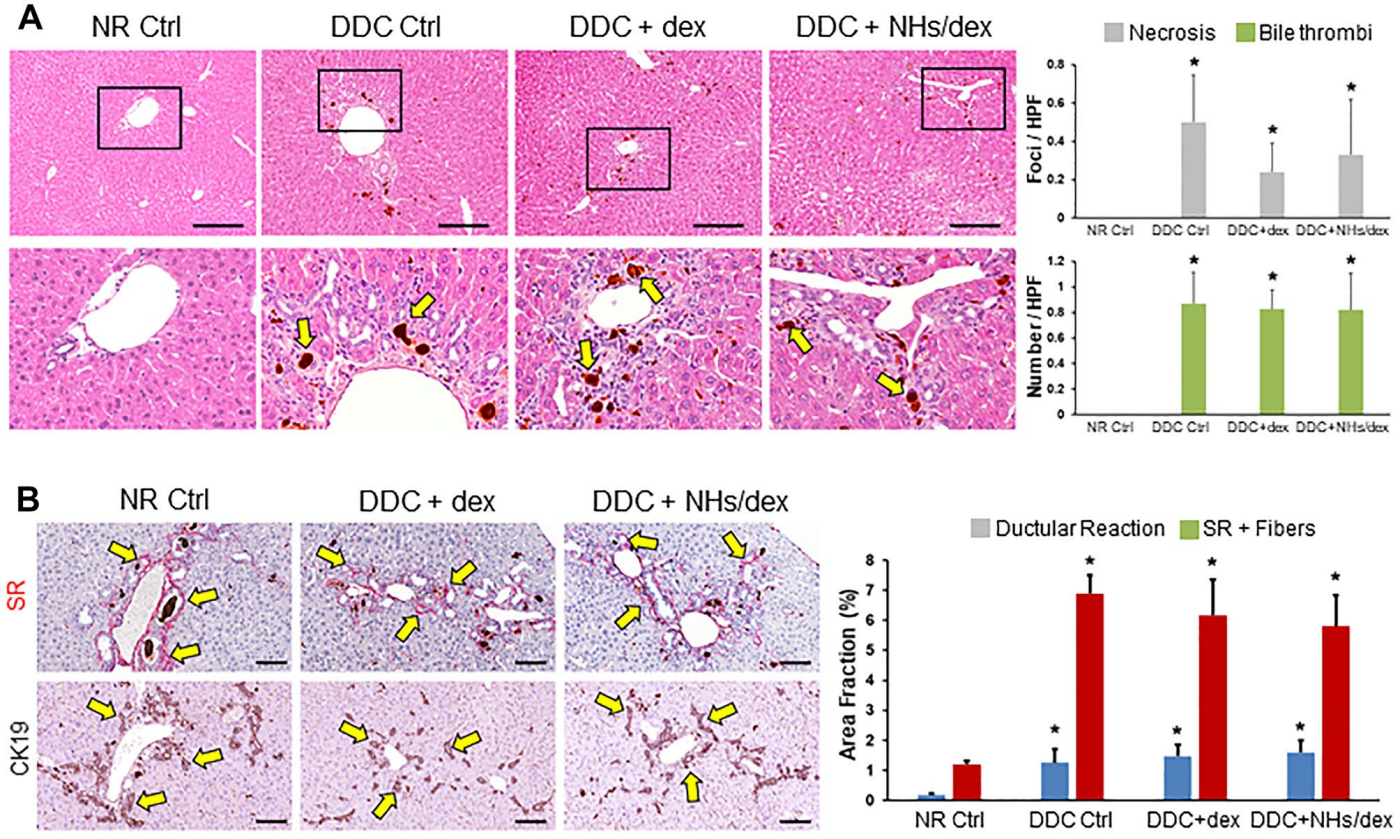
(**A**) Hematoxylin and eosin stains of murine livers. DDC diet induced liver necrosis, ductular reaction and bile thrombi (arrows). Necrosis is slightly reduced in mice treated with dex and NHs/dex, even this reduction was not statistically significant. Scale bar = 200 µm. Areas in the boxes are magnified below. (**B**) Sirius red stains and immunohistochemistry for CK19 in murine livers. DDC mice showed biliary fibrosis characterized by SR + collagen fibre deposition (in red, arrows) at portal levels and CK19 + ductular reaction (in brown, arrows). No significant differences were present after dex and NHs/dex treatments. Scale bar = 100 µm. *p < 0.05 versus normal (NR) mice

Mice subjected to DDC diet (DDC + Ctrl) showed histologic alterations in the liver parenchyma including hepatocyte necrosis, bile thrombi, ductular reaction, and periportal fibrosis (Fig. 6). Mice under control diet (Normal + Ctrl group) showed no histological modifications. The groups of DDC + dex or DDC + NHs showed histologic alterations similar to DDC + Ctrl. Mice treated with dex and NHs/dex showed a lower number of necrotic foci compared to DDC + Ctrl mice, but this reduction did not reach statistical significance (Fig. 6A). No differences were present in term of ductular reaction and fibrosis in DDC mice irrespectively to the treatment regimen (Fig. 6B).

## Discussion

The main results of this paper showed that NHs and NHs/dex are non-toxic in vitro for human cells and in a mouse model. Primary and immortalized cholangiocytes treated with NHs/dex show an increase in the functional marker expression of NHE1 cholangiocytes compared to control groups. A mouse model of cholangiopathy treated with NHs/dex shows a reduction in markers of hepatocellular injury compared to control groups (NHs, dex, or sham group).

The ability of HA-CH to form self-assembled NHs after autoclaving might be due to the establishment of intra- and inter-molecular forces among the moieties of similar nature in aqueous environment, which represent the driving forces for the self-aggregation of the polymer chains, thanks to the reduction of the interfacial free energy of the whole system [22, 42]. The assembly of HA-CH chains at 121 °C leads to the formation of nanoparticles with internal hydrophobic domains, which do not interact with the surrounding aqueous medium, and a hydrophilic outer layer, facing the solvent. CH was selected as a lipophilic grafting molecule of HA, even though its steroidal structure, characterized by high steric hindrance, does not allow direct esterification. To overcome this drawback, a short-chain fatty acid spacer of butyric acid was placed between the HA chains and the CH moieties, hence contributing to the formation of the NHs lipophilic domains. Previous studies showed that high HA derivatization degrees led to the formation of insoluble derivatives that did not form NHs. Thus, a DD of 15% mol_CH_/mol_HA_ was chosen to allow the NHs formation.

As previously reported, autoclaving at 121 °C and 1.1 bar for 20 min represents a suitable, fast and reproducible method to directly prepare sterile and drug-loaded NHs in a single step [42]. Thus, dexamethasone was loaded into NHs with very good reproducibility with this approach, as previously reported for the entrapment of other hydrophobic drugs. The prepared NHs/dex suspension showed a good stability at 4 °C, as well as in simulated physiological conditions, in terms of size and PDI. Besides, they were stable at 37 °C over 48 h, when a degradation in vivo is expected. Furthermore, the negative ζ-potential value of HA-CH NHs confers high stability to the formulation, thus preventing particle aggregation. Finally, to assess a long-life storage of NHs/dex formulation, drug-loaded NHs were freeze-dried and reconstituted with double-distilled water before use.

The effects of NHs/dex formulation were assessed in vitro in human cholangiocytes, namely the well-known H69 established human cholangiocytes cell line [43], and in primary cultures of human cholangiocytes derived from differentiation of hBTSCs [44]. These cultures were often used in previous studies to investigate biliary physiology and disease modelling [34, 35, 43, 45]. The obtained results showed that NHs/dex treatment significantly increases cell proliferation compared to dex. This unexpected result found a rationale in the recent demonstration of a novel pathway regulating LGR5^+^ epithelial stem cell proliferation and normal intestinal elongation in mouse [46]. In this pathway, endogenous extracellular hyaluronic acid binds to Toll-like receptor 4 inducing the release of PGE2, which binds to epidermal growth factor receptor on LGR5^+^ stem cells, thus leading to their proliferation [46]. Although this innovative pathway needs to be investigated in depth in the biliary tree, TLR4 was already found expressed in human cholangiocytes [35, 45].

To test the effects of dexamethasone delivered through the NH particles in vivo, the well-characterized DDC model was used in mice [47]. Chronic DDC diet has been proposed as an in vivo model for cholestatic disease due to the formation of intraductal porphyrin plugs. Chronic feeding of DDC in mice reproduces the main histopathological hallmarks of human cholestatic disease such as (1) remodelling of biliary compartments giving rise to ductular reaction, (2) periductular fibrosis, and (3) inflammatory infiltrate [47]. The universally recognized cholestatic biomarker, and useful endpoint in defined cholangiopathies like PBC, the AlkP, has been tested in mice treated for 4 weeks with DDC to induce bile duct injury and subsequently treated with dexamethasone alone or delivered through the NH particles for 17 days. While the mice of DDC + NHs, DDC + dex and DDC Ctrl groups showed significantly high levels of AlkP and ALT compared to Normal Ctrl group, the DDC + NHs/dex group had AlkP and ALT statistically lower compared to the other DDC groups. In particular, the amelioration of well-known serum marker of cholestasis, AlkP, and liver injury, ALT, is associated with an increase NHE-1 relative gene expression in murine liver in DDC + NHs/dex mice vs other experimental groups.

Intrahepatic biliary epithelium exerts a pivotal barrier function against the harsh environment of the bile mainly due to an active bicarbonate excretion, the so-called protective “bicarbonate umbrella” [2, 3]. The activity of the Cl-/HCO3- exchanger in cholangiocytes is balanced by acid extruders, namely the NHE-1 Na^+^/H^+^ exchanger isoform 1 and the Na^+^/HCO_3_^−^ cotransporter [4, 5].

The NHE-1 expression has been investigated by RT-qPCR in H69 and hBTSCs-CM primary cultures after a stimulation of 48 h. In both H69 and hBTSCs-CM, the gene expression of NHE-1 was significantly increased by NHs/dex compared to other treatments and Ctrl. It was previously demonstrated that the intrahepatic biliary epithelium expresses glucocorticoid receptors (GcRs) and responds to dexamethasone by increasing bicarbonate excretion in bile [6]. This is caused by corticosteroid-induced enhanced activities and protein expression of transport processes, Na^+^/H^+^ (NHE1) and Na^+^/HCO_3_^−^ exchangers (AE2), that driving bicarbonate excretion in the biliary epithelium [6].

The results suggest a rapid entrance and an optimized intracellular distribution of dexamethasone delivered through the NH particles. A possible pharmacokinetic explanation for the in vivo results may rely on the previously demonstrated liver uptake of the HA (90% actively cleared by the liver). [48]. Thus, dexamethasone delivered through the NHs could be actively cleared by the liver. HA recognizes a cognate receptor, the CD44, which is a structural protein involved in cell adhesion, interaction with extracellular matrix, cell migration and inflammatory signaling [48]. Finally, it could be affirmed that the use of NHs/dex formulation leads to an increased choleretic effect. Moreover, HA has opposite properties depending on the chain lengths and on the ability to bind to multiple CD44 receptors. Long-chains HA (HA-l) exhibit anti-inflammatory properties in many in vitro and in vivo models [48]; other studies reported that HA-l increase the phagocytosis by macrophages, reduce the pro-inflammatory cytokine production and limit cell oxidative damage and apoptosis [48]. Notably, NHs used in this study fall under the definition of HA-l, being its molecular weight > of 100.000 g/mol.

In addition, the available commercial HA complies with cGMP manufacturing requirements and is approved for clinical use (in particular, for osteoarticular, ocular and cutaneous damages) [49, 50]. Furthermore, 90% of HAs are actively cleared by the liver [48, 51–53].

To date, the therapeutic options for the treatment of cholestasis remain limited. Several works showed that intrahepatic cholestasis of different origins may transiently improve after a short treatment with corticosteroids [6]. However, the use of steroids is strongly limited by their side effects, mainly the immune-suppression and infective risk and the glucocorticoid-induced osteoporosis. In this context, nanoparticles may represent a useful tool in the treatment of this pathology as they can be engineered to directly deliver steroids to the target location, thus limiting their systemic side effects. The novelty of this work is related to the use of hyaluronan-based NHs as a carrier for the delivery of the corticosteroid dexamethasone. In fact, the overexpression of CD44 receptors during liver injury can allow NHs/dex to selectively accumulate in the injured liver, leading to an in situ release of the dex, while, at the same time, HA may alleviate the oxidative/inflammatory-induced damage. Furthermore, in vivo studies confirmed that NHs/dex formulations may be more effective in the treatment of cholestasis compared to free dex, opening the route for the use of HA-based NHs for the treatment of liver injuries. Being the present work, the first study investigating the NHs/dex combination in experimental cholestasis, preclinical studies were carried out.

In particular, in established and primary human cholangiocytes cell lines, it was noted a significant (although not dramatic) increase of cell proliferation in the presence of NHs/dex or NHs. This result may be indicative of a potential cancerogenicity of the systems. Although long term experiments in vivo were not performed, to obtain conclusive safety data, no differences were present in terms of ductular reaction (expression of proliferation of cholangiocyte and oval cells in murine liver) and fibrosis in DDC mice, regardless of the treatment regimen. Another aspect that needs to be investigated more in detail is the effect of NHs/dex on the different liver cell populations, epithelial and not epithelial (macrophages, stellate cells, endothelial cells, etc.). The effect of the NHs/dex formulation deserves to be studied in depth on the multiple types of liver cells.

## Conclusions

One of the major limitations of giving steroids is represented by their systemic adverse events. Even a corticosteroid with a high receptor affinity and hepatic first pass clearance, like budesonide, may exert systemic unwanted effects, being particularly detrimental in patients with cholangiopathies, e.g. glucocorticoid-induced osteoporosis [9], or with advanced liver diseases, e.g. immune-suppression and infective risk [10, 32, 54–56]. Thus, increasing the delivery of steroids to the liver may result in two beneficial effects: the first one on the treatment of autoimmune or inflammatory liver damages [55, 56] and the second one on the reduction of the steroid systemic harmful effects [9, 10].

Our results show that NHs and NHs/dex are non-toxic in vitro for human cells and in vivo for mouse models. Primary and immortalized cholangiocytes treated with NHs/dex show an increase in the functional marker expression of NHE1 cholangiocytes compared to control groups. A mouse model of cholangiopathy treated with NHs/dex shows a reduction in markers of hepatocellular injury compared to control groups (NHs, dex or sham group).

In conclusion, we believe that the NHs/dex formulation is a suitable candidate to be investigated in preclinical models of cholangiopathies.

## Data Availability

All data relevant to the study are included in the article.
